# Supplementary material for: Transition experiences of patients with post stroke dysphagia and family caregivers: A longitudinal, qualitative study
Source: PLoS One. 2024 Jun 4;19(6):e0304325. doi: 10.1371/journal.pone.0304325 (PMC11149836; doi:10.1371/journal.pone.0304325)
Supplement: S1 Checklist — (DOCX) [file pone.0304325.s001.docx]

**S1 Checklist:**

**Consolidated criteria for reporting qualitative studies (COREQ): a 32-item checklist**

Developed from:

Tong A, Sainsbury P, Craig J. Consolidated criteria for reporting qualitative research (COREQ): a 32-item checklist for interviews and focus groups. *International Journal for Quality in Health Care*. 2007. Volume 19, Number 6: pp. 349 – 357.

| **No. Item** | **Guide questions/description** | **Reported on Page #** |
| --- | --- | --- |
| **Domain 1: Research team and reflexivity** | | |
| *Personal Characteristics* | | |
| 1. Interviewer/facilitator | Which author/s conducted the interview or focus group? | Page 7-8: Data collection |
| 1. Credentials | What were the researcher’s credentials? E.g. PhD, MD | Postgraduate student |
| 1. Occupation | What was their occupation at the time of the study? | Researchers |
| 1. Gender | Was the researcher male or female? | Female |
| 1. Experience and training | What experience or training did the researcher have? | Page 9: Rigour |
| *Relationship with participants* | | |
| 1. Relationship established | Was a relationship established prior to study commencement? | Page 9: Rigour |
| 1. Participant knowledge of the interviewer | What did the participants know about the researcher? e.g. personal goals, reasons for doing the research | Page 9: Ethics |
| 1. Interviewer characteristics | What characteristics were reported about the interviewer/facilitator? e.g. Bias, assumptions, reasons, and interests in the research topic | Page 9: Rigour |
| **Domain 2: study design** | | |
| *Theoretical framework* | | |
| 1. Methodological orientation and Theory | What methodological orientation was stated to underpin the study? e.g. grounded theory, discourse analysis, ethnography, phenomenology, content analysis | Page 6: Study design |
| *Participant selection* | | |
| 1. Sampling | How were participants selected? e.g. purposive, convenience, consecutive, snowball | Page 6-7: Participants and settings |
| 1. Method of approach | How were participants approached? e.g. face-to-face, telephone, mail, email | Page 7-8: Data collection |
| 1. Sample size | How many participants were in the study? | Page 10-11: Findings |
| 1. Non-participation | How many people refused to participate or dropped out? Reasons? | Page 10-11: Findings |
| *Setting* | | |
| 1. Setting of data collection | Where was the data collected? e.g. home, clinic, workplace | Page 7: Participants and settings |
| 1. Presence of non-participants | Was anyone else present besides the participants and researchers? | Page 8: Data collection |
| 1. Description of sample | What are the important characteristics of the sample? e.g. demographic data, date | Page 10-11: Findings and Table 1. |
| *Data collection* | | |
| 1. Interview guide | Were questions, prompts, guides provided by the authors? Was it pilot tested? | Page 8: Data collection |
| 1. Repeat interviews | Were repeat interviews carried out? If yes, how many? | Yes. Page 10-11: Table 1 . |
| 1. Audio/visual recording | Did the research use audio or visual recording to collect the data? | Page 8: Data collection |
| 1. Field notes | Were field notes made during and/or after the interview or focus group? | Page 8: Data collection |
| 1. Duration | What was the duration of the interviews or focus group? | Page 8: Data collection |
| 1. Data saturation | Was data saturation discussed? | Page 10-11: Findings |
| 1. Transcripts returned | Were transcripts returned to participants for comment and/or correction? | Page 9: Rigour |
| **Domain 3: analysis and findings** | | |
| *Data analysis* | | |
| 1. Number of data coders | How many data coders coded the data? | Page 10: Data analysis |
| 1. Description of the coding tree | Did authors provide a description of the coding tree? | No. |
| 1. Derivation of themes | Were themes identified in advance or derived from the data? | Yes, page 12-19. |
| 1. Software | What software, if applicable, was used to manage the data? | No. |
| 1. Participant checking | Did participants provide feedback on the findings? | No, not specifically. |
| *Reporting* | | |
| 1. Quotations presented | Were participant quotations presented to illustrate the themes/findings? Was each quotation identified? e.g. participant number | Yes, page 12-19. |
| 1. Data and findings consistent | Was there consistency between the data presented and the findings? | Yes, page 12-23: Findings and Discussion |
| 1. Clarity of major themes | Were major themes clearly presented in the findings? | Yes, page 12-19: Findings and Figure 2. |
| 1. Clarity of minor themes | Is there a description of diverse cases or discussion of minor themes? | Yes, page 12-23: Findings and Discussion |
