# Supplementary material for: Transition experiences of patients with post stroke dysphagia and family caregivers: A longitudinal, qualitative study
Source: PLoS One. 2024 Jun 4;19(6):e0304325. doi: 10.1371/journal.pone.0304325 (PMC11149836; doi:10.1371/journal.pone.0304325)
Supplement: S1 File — (DOC) [file pone.0304325.s002.doc]

Topic guide informed by the main concepts of Meleis’s Transition Theory

- Topic guide during hospitalization

| **Main concepts of Meleis’s Transitions Theory** | **Interview questions** |
| --- | --- |
| Nature of transitions | 1.Could you tell me something about your hospitalization experience? How do you feel? |
|  | 1. How has your diet changed since you were hospitalized? (Tips: the way of eating, the choice of food, the speed of eating, the amount of food eaten, etc.)   3.What is the impact of dysphagia on your life and family? (Tips: diet, social interaction, etc.) |
| Transition conditions of personal | 4.Have you learnt something about dysphagia during your hospitalization? By what means? By whom? |
| Transition conditions of community  Patterns of response: process indicators | 5.What support and assistance did your family, relatives or friends provide during this period? |
|  | 6.Did you encounter any difficulties during admission and hospitalization? If so, which ones? Who helped you solve these difficulties? How was it solved? |
| Nursing therapeutics | 8.What treatment and care have you had for dysphagia while in hospital? Which ones are the most effective? |

- Topic guide of telephone follow-up after discharge

| **Main concepts of Meleis’s Transitions Theory** | **Interview questions** |
| --- | --- |
| Nature of transitions | 1.Can you talk about your/the patient’s experience after being transferred or discharged home? How did you feel after being transferred to the hospital or home? |
|  | 2.How did you feel about dysphagia care after discharge? (Tips: eating patterns, food choices, tube care, etc.) |
|  | 3.Has dysphagia affected your/the patient's life and family after discharge from the hospital? What are the specifics? (diet, social interactions, etc.) |
| Transition conditions of personal | 4.Have you learnt something about dysphagia care after transfer/discharged home? In what ways? By whom? |
| Transition conditions of community  Patterns of response: process indicators | 5.Have you received help from others since being discharged from the hospital? (Tips: family or relatives, friends, community agencies, etc.) |
|  | 6.Did you have any difficulties with the transfer/discharge home process? If so, which ones? Who helped you/the patient to resolve these difficulties? How were they resolved? |
| Nursing therapeutics | 7.Have you/the patient received dysphagia related rehabilitation training after transfer or discharge home? What training methods are there? Which ones have you found to be the most effective? |
