# Supplementary material for: Transition experiences of patients with post stroke dysphagia and family caregivers: A longitudinal, qualitative study
Source: PLoS One. 2024 Jun 4;19(6):e0304325. doi: 10.1371/journal.pone.0304325 (PMC11149836; doi:10.1371/journal.pone.0304325)
Supplement: S2 File — (DOC) [file pone.0304325.s003.doc]

**Themes and associated comments.**

| **Themes** | **Subthemes** | **Participants Comment** |
| --- | --- | --- |
| Transition from onset to admission | Stroke occurred in an unexpected and abrupt way | P4: “After I woke up to wash my face, I saw that my face was different. I wanted to speak, but I couldn’t say anything, and my hands couldn’t lift.” |
|  |  | F7: “It happened so suddenly. We were having dinner, and we saw that my husband couldn’t walk well and couldn’t speak clearly.” |
|  |  | F3: “We went to wake him up for breakfast and all of a sudden we realized his mouth was crooked.” |
|  |  | F4: “The day before, he was fine and rode a motorcycle. I don’t know what happened, but suddenly he became like this. He couldn’t speak or eat.” |
|  | Inadequate dysphagia related knowledge and information | F4: “He was not so serious on the day of onset. We thought he just choked, but now he couldn’t eat anything.” |
|  |  | P2: “At first I thought I just had no appetite, and it was common to choke when you were old. However, look at me now, I couldn’t even drink water.” |
|  |  | F8: “I’ve heard of stroke before, but I haven’t specifically learned about dysphagia. If it weren’t for this onset, I wouldn’t have known about it.” |
|  |  | F2: “The doctor said that she has dysphagia, but we would rather eat slowly than inserting a gastric tube. I think the most difficult part is what kind of food we can choose, and we are afraid that his nutrition can’t keep up.” |
|  |  | P5: “Neither of us is a professional, and since I can’t eat by myself, I’m more worried that I won’t be able to handle it when I get home.” |
|  |  | F10: “It is scary when my uncle drinks water because he sometimes chokes badly. His face will turn purple and we don’t know what to do. So we hope that medical employees can teach us how to deal with choking before leaving the hospital.” |
|  |  | F6: “Now he can’t eat by himself, and he can only get food from the stomach tube. If we have to keep the tube when we leave the hospital, how can we take care of it at home? If the tube is pulled out, can we plug it back in ourselves?” |
|  |  | F11: “Is it possible to replace this tape for fixing the stomach tube with something else? He has to put this tape on every day, which makes his face red all over. I’m worried that it will break his skin. Is there any other way?” |
|  | Cooperate with swallowing rehabilitation training actively | P4: “I want to recover as soon as possible, especially my swallowing function, otherwise you can’t enjoy a lot of things. The food is injected directly into the stomach, and even the best things have no feeling.” |
|  |  | P6: “My family has worked hard to take care of me. I will cooperate with all treatment and training I need. I want to recover soon.” |
|  |  | F6: “I hope they can help my grandma get her tubes out soon, it sure would be nice to be able to eat on her own.” |
|  |  | F10: “We heard that acupuncture can also help to restore swallowing function. Can you give him this rehabilitation method?” |
|  | Impact of dysphagia | P5: “You can’t enjoy a lot of things anymore. If you just pour it directly into your stomach, you won’t feel anything even if it’s the best thing.” |
|  |  | P4: “I used to speak in a clear and melodious voice, but now it’s like I were thirty years older. I loved to sing, look at this voice now, I can’t sing anymore.” |
|  |  | F8: “I’ve been taking care of him since he got sick. I can't leave him alone. I’m afraid he’ll take the tube out.” |
|  |  | F3: “I had already found a job, but he (the patient) had a sudden attack and needed someone to take care of him. So I didn’t go back to work.” |
| Transition from discharge to other rehabilitation institutions | Incomplete handover of medical information and lack of information sharing platform | F7: “We already had a swallowing assessment done at the hospital before we were transferred, it failed. And after we were transferred, the nurses here did it again, and it still failed. so why do we have to do it all over again? I was afraid that he(the patient) would choke.” |
|  |  | F6: “I felt that swallowing rehabilitation training was quite good in your hospital before, but after the transfer, the doctor here gave she(patient) a new training method, so I was a little worried. What if this rehabilitation effect is not as good as before?” |
|  |  | F11: “You should have a special person in charge of the handover when we transfer to another hospital. It’s safe to have professional people with us, especially these with tubes such as gastric tube, endotracheal intubation.” |
|  | Gap in swallowing rehabilitation nursing services between hospitals | F8: “We just hope that my dad can be cured in your hospital, and we shouldn’t be transferred to other hospitals. We heard that your hospital is the best hospital for cerebrovascular diseases. Will the risk be higher if my dad is transferred out?” |
|  |  | P1: “I can’t see my attending doctor every day here. The doctors in your hospital are very detailed, that is, every doctor knows the condition very well, which may not be the case here.” |
|  |  | F1: “I feel that the nurses here are not so professional. I don’t trust them very much.” |
|  | Differences in ward environments | P4: “It’s a good environment here, not as crowded as the hospital before. I can do some training back and forth in the corridor.” |
|  |  | P2: “The wards in lower-level hospitals feel more spacious and quieter, which is suitable for recuperation.” |
| Transition from discharge to home | Continuous nursing services for dysphagia | P1: “When she(the patient) was discharged, the nurse told us a lot of precautions, but what she(the nurse) said was too much for us to remember, and when we got home. I think the WeChat follow-up visit is very good, but sometimes there was no reply when asked.” |
|  |  | F10: “Most importantly, we’re worried about his swallowing function. It would be nice if you could tell us when it will recover.” |
|  |  | F7: “I still have a question now, that is, whether he(the patient) will recover or not. The doctor said it may take a long time to recover, but how long will it take?” |
|  |  | P3: “After coming back from the hospital, my wife is the one who takes care of me. There are only us two at home, and we haven’t learned professional knowledge. Sometimes we don’t know whether we have done it right or not. I hope you can give more guidance in this respect.” |
|  |  | P5: “For example, you said I needed a high-protein diet, but we didn’t know what high-protein foods were. You should arrange nurses to teach us, like some precautions and so on.” |
|  | Lack of specialized swallowing rehabilitation training institutions | F5: “You know, we want to go to your hospital for rehabilitation training, but we haven’t been able to make an appointment, and we don’t want to go to other hospitals. What should we do?” |
|  |  | F11: “At that time, he had not fully recovered, and we were still worried after he was discharged from the hospital. It was more troublesome to come back for a follow-up visit and do rehabilitation training, for example, we waited for several days to reserve a bed.” |
|  |  | P2: “We don’t know how to do rehabilitation training at home, it would be nice to have a professional to guide us.” |
|  | Psychological changes in the long-term rehabilitation process | P2: “I’ve given up now, it’s been so long, and it can’t be cured. Nothing has changed. Oh well, so be it...” |
|  |  | F1: “We take turns taking care of her at home. All of us have to go to work, and we all take care of her during our rest time, but she hasn’t been well for so long. We are truly tired(sobbing).” |
